# Real-time estimation of paracellular permeability of cerebral endothelial cells by capacitance sensor array

**DOI:** 10.1038/srep11014

**Published:** 2015-06-05

**Authors:** Dong Hyun Jo, Rimi Lee, Jin Hyoung Kim, Hyoung Oh Jun, Tae Geol Lee, Jeong Hun Kim

**Affiliations:** 1 Fight against Angiogenesis-Related Blindness (FARB) Laboratory, Clinical Research Institute, Seoul National University Hospital, Seoul, Republic of Korea; 2 Department of Biomedical Sciences, College of Medicine, Seoul National University, Seoul, Republic of Korea; 3 Center for Nano-Bio Measurement, Korea Research Institute of Standards and Science Daejeon, Republic of Korea; 4 Department of Ophthalmology, College of Medicine, Seoul National University, Seoul, Republic of Korea

## Abstract

Vascular integrity is important in maintaining homeostasis of brain microenvironments. In various brain diseases including Alzheimer’s disease, stroke, and multiple sclerosis, increased paracellular permeability due to breakdown of blood-brain barrier is linked with initiation and progression of pathological conditions. We developed a capacitance sensor array to monitor dielectric responses of cerebral endothelial cell monolayer, which could be utilized to evaluate the integrity of brain microvasculature. Our system measured real-time capacitance values which demonstrated frequency- and time-dependent variations. With the measurement of capacitance at the frequency of 100 Hz, we could differentiate the effects of vascular endothelial growth factor (VEGF), a representative permeability-inducing factor, on endothelial cells and quantitatively analyse the normalized values. Interestingly, we showed differential capacitance values according to the status of endothelial cell monolayer, confluent or sparse, evidencing that the integrity of monolayer was associated with capacitance values. Another notable feature was that we could evaluate the expression of molecules in samples in our system with the reference of real-time capacitance values. We suggest that this dielectric spectroscopy system could be successfully implanted as a novel *in vitro* assay in the investigation of the roles of paracellular permeability in various brain diseases.

Vascular integrity is essential for maintaining homeostasis of brain microenvironments^1^. Blood-brain barrier (BBB), of which main constituent cells are brain microvascular endothelial cells, act as “barriers” to regulate paracellular passage of molecules^2^,^3^. In various brain diseases including Alzheimer’s disease, stroke, and multiple sclerosis, pathological conditions is linked with compromised microvasculature integrity^2^,^4^. The increase in vascular permeability leads to uncontrolled flux of immune cells, molecules, and ions, resulting in neuronal dysfunction, neuroinflammation, and neurodegeneration^2^. Accordingly, it is required to restore vascular integrity to minimize the detrimental effects of increased vascular permeability. In this context, it is helpful to develop *in vitro* methods to measure the changes of brain microvascular endothelial cells for investigating the effects of certain factors on the integrity of cerebral microvasculature.

Currently utilized methods include the measurement of expression levels of tight junction proteins by immunofluorescent staining or immunoblot and electrical measuring methods, including electric cell-substrate impedance sensing (ECIS)^5^ and transendothelial electrical resistance (TEER)^6^. ECIS measures the alternating current (AC) impedance between a small sensing electrode and a large counter electrode while cells are cultured on the gold sensing electrode. In this system, attached cells spread on the surface of the sensing electrode and passively block the current to affect the electrode impedance by the shape, adhesiveness, and/or mobility of adherent cells^7^,^8^. On the other hand, TEER quantitatively measures the barrier integrity by electrical ohmic resistance (R) of the endothelial monolayer. In principle, it can be determined by a direct current (DC)-based approach: a defined DC voltage (U) is applied to two electrodes, each of which is placed on each side of the cell layer. By the measurement of the current (I), the ohmic resistance can be estimated according to Ohm’s law (R = U/I)^9^.

The capacitance (or dielectric constant) could be also a potential candidate to estimate the changes in the integrity of cerebral microvasculature. This method is a useful way to obtain real-time electrical properties such as cell membrane capacitance and cytoplasm conductivity^10^ Upon application of AC electrical fields, cells demonstrate specific dielectric responses according to interfacial polarization patterns^11^ Interestingly, recent studies have demonstrated that this technique can be utilized to observe various cellular functions including cell viability/death, endocytosis, and differentiation^10^,^12^,^13^,^14^,^15^ In the similar manner, we speculated that we could differentiate the status of paracellular permeability of cerebral microvascular endothelial cell monolayer with the capacitance sensor array.

In this study, we developed a capacitance sensor array which could measure real-time capacitance of 16 wells of endothelial cell monolayer at a time. Capacitance measurement in endothelial cells treated with vascular endothelial growth factor (VEGF), permeability-inducing factor, and its inhibitor demonstrated differential frequency- and time-dependent capacitance patterns according to the status of paracellular permeability. Interestingly, we could measure the alteration in dielectric responses as a result of increased or decreased tightness of junctions in endothelial cell monolayer. With this novel system, we could compare normalized capacitance values among different treatment groups. Furthermore, the status of tight junction proteins was evaluated through immunocytochemical staining with our capacitance sensor array after successful recording of capacitance values for 3 days. We suggest that this capacitance sensor array would be utilized to estimate paracellular permeability of cerebral endothelial cells.

## Results

### Capacitance sensor array measures frequency- and time-dependent changes in capacitance values of cerebral microvascular endothelial cells

**Figure 1:**
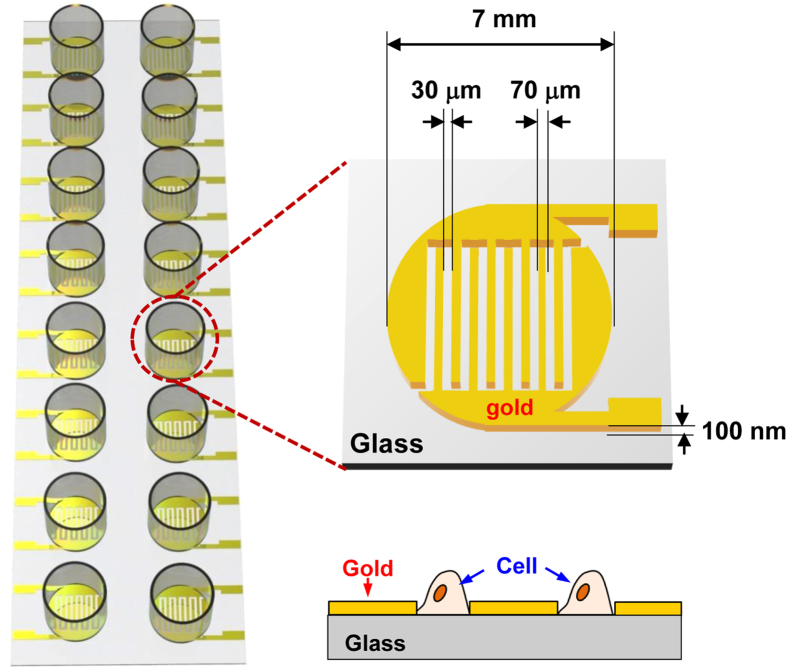
Schematic diagram of a capacitance sensor array to estimate paracellular permeability of cerebral endothelial cells in monolayer

We developed a capacitance sensor array consisting of 16 sensors with interdigitated electrodes (Fig. 1). The sensor was placed in the incubator supplemented with 95% air and 5% CO_2_ at 37 °C and was connected to the data acquisition unit which collected data from each sensor every 5 minutes (Supplementary Fig. 1). In our system, cells were plated between gold electrodes and the change in dielectric constant (ε) is measured to estimate the capacitance (*C*) with an equation *C *= ε*A*/*d*, where *A* is the electrode area and *d* is the distance between the two electrodes^12^,^13^,^14^,^15^. To increase paracellular permeability of cerebral endothelial cells, we treated endothelial cells in monolayer with VEGF (20 ng/mL), which is known to increase cerebral vascular permeability,^16^,^17^ after 24 hours of stabilization. Then, we observed the effect of the treatment of VEGF with or without anti-VEGF antibody for additional 48 hours (until 72 hours after the initial measurement). As in other studies, VEGF treatment induced down-regulation of tight junction proteins, which was reversed by cotreatment with anti-VEGF antibody (Supplementary Fig. 2).

In particular, we hypothesized that VEGF treatment would induce differential responses at low frequency; because, at the low AC frequency (few kHz), the membrane capacitance is high and most electrical currents pass through paracellular space. On the other hands, at high AC frequency (tens of kHz and tens of MHz), the capacitance of the membrane is relatively small and electrical currents mainly penetrate the insulating cell membranes with little current passing through paracellular path^10^,^18^.

**Figure 2:**
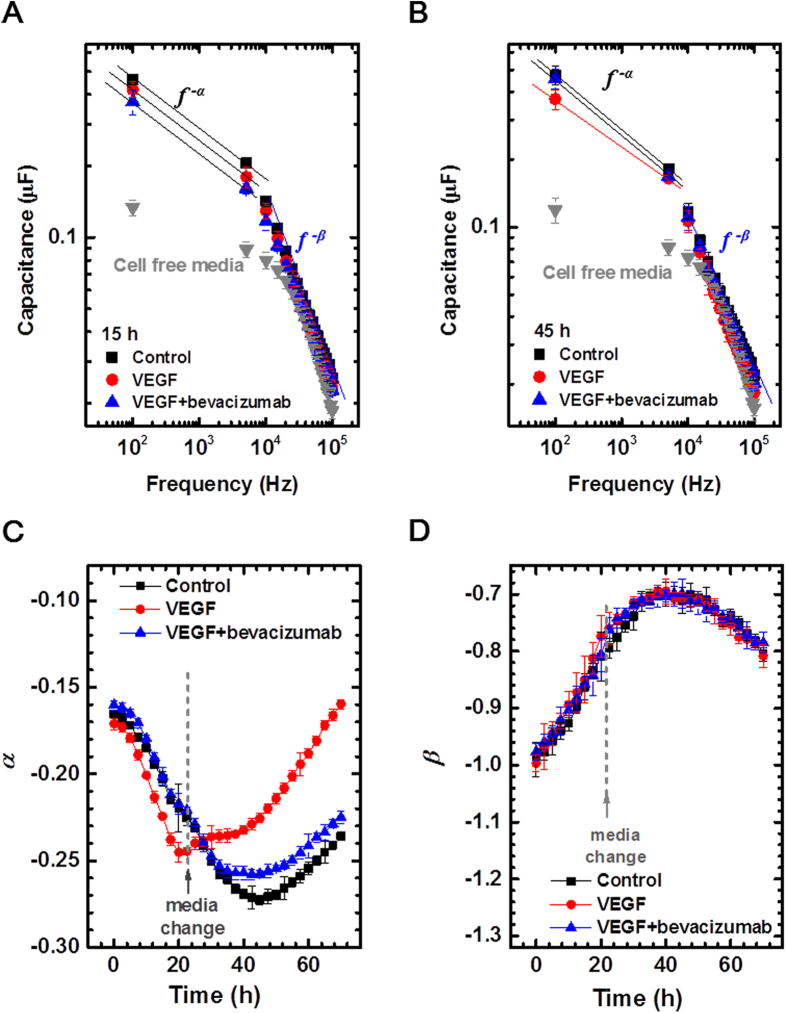
Frequency- and time-dependent capacitance measurement in cerebral microvascular endothelial cells. At 15 hours (**A**) and 60 hours (**B**) after the initial measurement, capacitance was measured at various frequencies from 100 Hz to 100 kHz (*n *= 3). Capacitance readings were fitted to fitting lines in a relationship of *C* ∝ *f*^−α^ and *C* ∝ *f*^−β^ in the frequency range from 100 Hz to 5 kHz and from 75 kHz to 100 kHz, respectively. (**C**, **D**) Time-dependent estimates of *α* (**C**) and *β* (**D**) from real-time capacitance measurement using a capacitance sensor array.

To determine whether the status of endothelial cell monolayer could be discriminated according to the treatment of VEGF with or without anti-VEGF antibody using our capacitance sensor, we measured capacitance values as a function of frequency (*f*) in cerebral microvascular endothelial monolayer (*n* = 3, Fig. 2A,B). At 15 hours after the initial measurement (before treatment), the capacitance was fitted to the relationship of *C* ∝ *f*^−α^ with α ≈ 0.20 ~ 0.22 (Fig. 2A) for all three groups. In contrast, at 60 hours after the initial measurement (36 hours after the treatment of VEGF with or without anti-VEGF antibody), the capacitance values followed the relationship of *C* ∝ *f*^−α^ with α ≈ 0.24 ~ 0.25 in the control group and the group with co-treatment of VEGF and anti-VEGF antibody, whereas with α ≈ 0.19 for the group with VEGF treatment only at low frequencies (Fig. 2B). At high frequencies, when the capacitance values were fitted to *C* ∝ *f*^−β^, β was estimated to be 0.86 at 15 hours and 0.74 ~ 0.75 at 60 hours after the measurement in all groups. For confirmation of these observations, we measured the frequency-dependent capacitance in ARPE-19 and human retinal microvascular endothelial cells (HRMECs) at various time points. These cells constitute inner and outer blood-retinal barrier as brain microvascular endothelial cells are components of BBB. Interestingly, similar behaviours were observed in both cells with the treatment of VEGF and tumour necrosis factor-α (TNF-α) treatment as brain microvascular endothelial cells, although the exact capacitance values were different according to the cell lines and time points (Supplementary Fig. 3,4).

Furthermore, we evaluated time-dependent changes of *α* and *β* according to the treatment groups. A remarkable finding was that VEGF treatment resulted in distinguishing pattern of *α* value compared to those demonstrated in control and VEGF plus bevacizumab groups (Fig. 2C). Unlike the latter 2 groups which showed a dip around 44 hours after the initial measurement, VEGF treatment induced gradual increase in α value after the treatment. In contrast to differential patterns in *α* values, variations in *β* values were quite similar with each other in patterns; they had a slight peak at around 44 hours after the initial measurement with adjacent plateaus (Fig. 2D).

### Capacitance values can be normalized to demonstrate differential levels according to paracellular permeability

**Figure 3:**
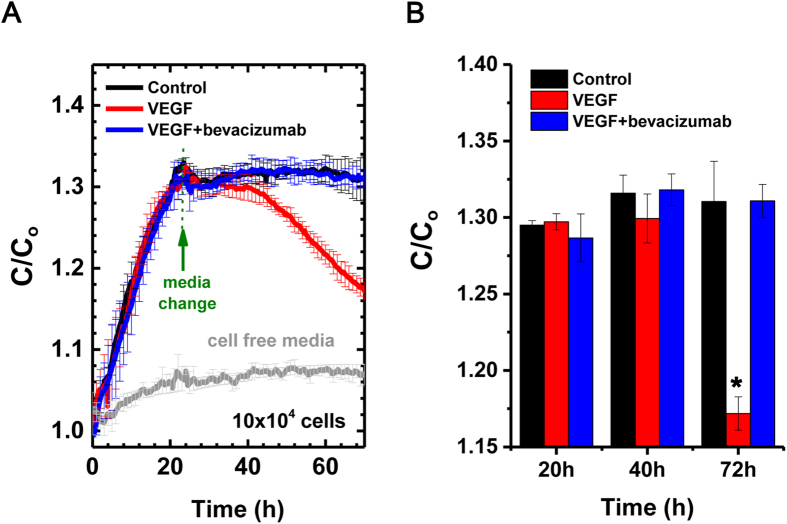
Normalized capacitance values in cerebral microvascular endothelial cells according to paracellular permeability. (**A**) Capacitance values were normalized using C_0_, the capacitance measured from cell-free media at Day -1 (*n *= 5). (**B**) Bar graph of normalized capacitance values at 20 hours, 40 hours, and 72 hours after the initial measurement. *, *P *< 0.01 (two-tailed).

Next, the real-time capacitance values at 100 Hz were measured in three treatment groups (*n* = 5, Fig. 3A). The measured capacitance values (C) were normalized to the initial values (C_0_) since C_0_ values were slightly different from different sensors. Interestingly, there was no definite change in patterns and absolute values between control and VEGF plus bevacizumab groups. In contrast, VEGF treatment induced definite change in capacitance values from the other groups. The normalized values started to drop at around 44 hours after the initial measurement and rapidly decreased after 48 hours from the initial measurement, resulting in significant difference in the normalized values at 72 hours after the initial measurement (*P* < 0.01; Fig. 3B). In this manner, we could quantitatively analyse the change in endothelial cell monolayer by the results of capacitance measurements.

Similar results were observed in HRMECs and ARPE-19 cells, retinal microvascular endothelial cells and retinal pigment epithelial cells, respectively. There were no definite changes in patterns and absolute values between control and VEGF plus bevacizumab groups in HRMECs and between TNF-α plus anti-TNF-α antibody groups in ARPE-19 cells. However, VEGF or TNF-α treatment induced definite change in values from the other groups. The normalized values started to drop at around 35 hours in case of HRMECs and 32 hours for ARPE-19 cells and rapidly decreased after 39 and 34 hours from the initial measurement, resulting in significant difference in the normalized values at 72 hours after the initial measurement (P < 0.01; Supplementary Figs 3C,D and 4C,D).

### Capacitance sensor array reflects the status of monolayer culture of cerebral microvascular endothelial cells: sparse or confluent conditions

**Figure 4:**
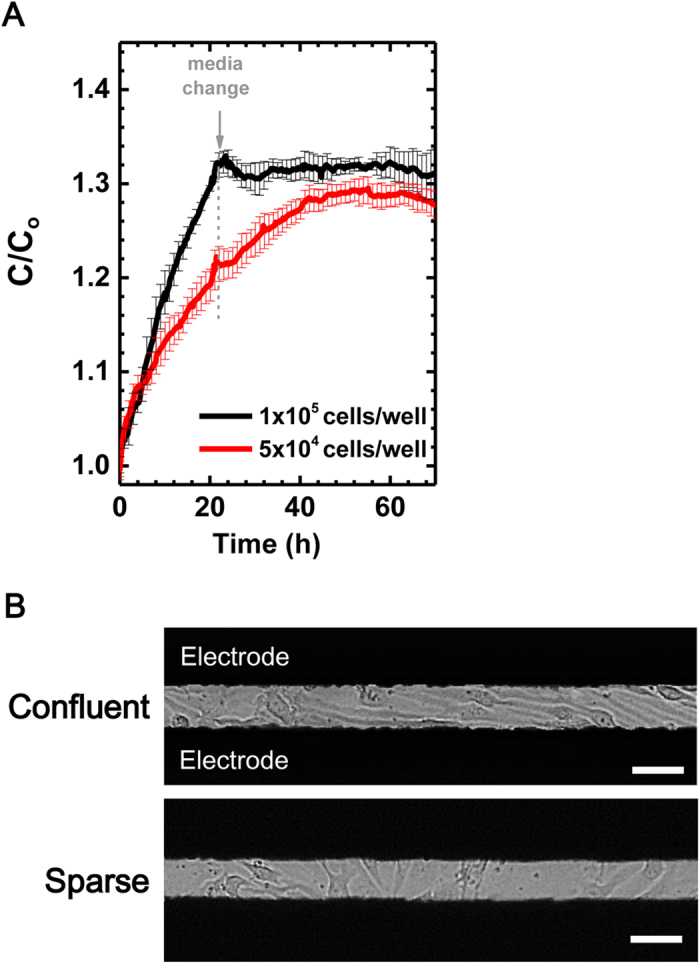
Capacitance measurement of cerebral endothelial cells according to the status of monolayer, confluent or sparse. (**A**) Normalized capacitance values in two different culture conditions. (**B**) Optical images of bEnd.3 cells in confluent or sparse culture conditions. Scale bars, 50 μm.

Another notable feature in our system was that the status of monolayer culture of cerebral microvascular endothelial cells could be evaluated by direct observation of cells and normalization of capacitance values. The patterns of normalized capacitance values from cerebral endothelial cells in confluent and sparse culture conditions were definitely different (Fig. 4A,B). Interestingly, well-prepared endothelial cell monolayer with adequate cell numbers demonstrated stable capacitance values from 24 hours to 72 hours after the initial measurement (Fig. 4A). We speculated that stable formation of cell-cell contacts might regulate electrical properties of monolayer and induce stable responses to extrinsic AC currents at low frequency. In conventional TEER system, endothelial cells should be prepared as confluent to measure permeability changes by resistance. In contrast, capacitance sensors focus cell-to-cell membrane contact and paracellular current flow. The permeability changes can be observed in even sparse culture conditions. Similar patterns were observed in HRMECs and ARPE-19 cells (Supplementary Figs 3C,E and 4C,E).

### Capacitance values are related with the status of tight junction complexes demonstrated by immunofluorescent staining of tight junction proteins

**Figure 5:**
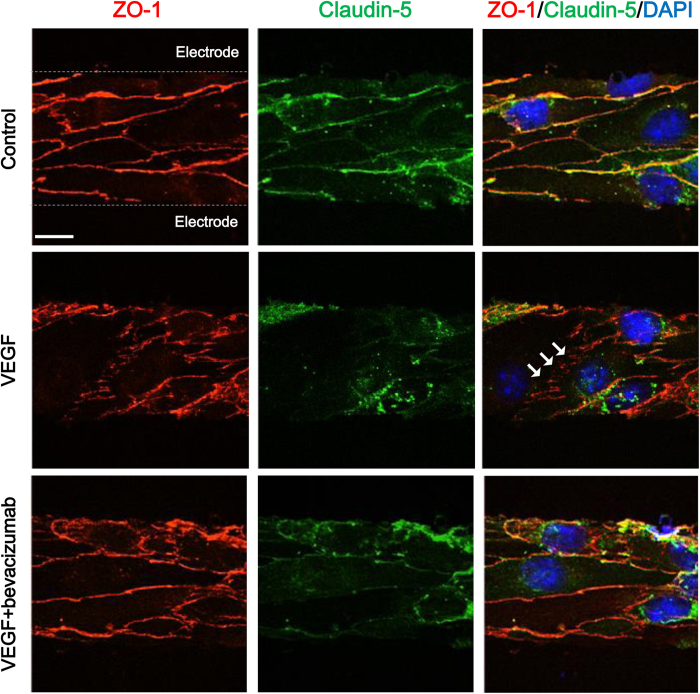
Immunocytochemical staining of tight junction proteins in cerebral endothelial cells with different paracellular permeability. White arrows indicate disrupted alignment of tight junction proteins. Scale bars, 10 μm.

After the measurement of capacitance values, we fixed cells with 4% paraformaldehyde and performed immunofluorescent staining of ZO-1 and claudin-5. These two proteins were selected as representative proteins in tight junction complexes of BBB. As shown in Fig. 5, VEGF treatment induced disturbances in alignment of tight junction proteins (white arrows in Fig. 5). In contrast, bevacizumab cotreatment effectively reversed detrimental effects of VEGF on the tight junction complexes, demonstrating well-aligned distribution of tight junction proteins along the cell membranes. We also observed similar results in HRMECs and ARPE-19 cells (Supplementary Fig. 5). In this way, we could easily examine the expression of tight junction proteins in our system after successful recordings of capacitance values.

## Discussion

In this study, we measured real-time capacitance values from 16 sensors with interdigitated electrodes in our capacitance sensor array. Every 5 minutes, each sensor captured electrical properties of cerebral endothelial cell monolayer. We could analyse time- and frequency-dependent capacitance changes from these real-time measurements. With interdigitated electrodes, capacitance values were obtained from cells between electrodes in a relationship of *C *= ε*A*/*d* without additional manual electrodes in conventional ECIS system. Interestingly, dielectric spectroscopy is a useful tool to monitor cellular events such as cell death, cell differentiation, and endocytosis^10^,^12^,^13^,^14^,^15^. In line with these studies, we showed that capacitance measurement could also be utilized in monitoring paracellular permeability of cerebral endothelial cell monolayer.

Interestingly, this system could evaluate the status of monolayer, confluent or sparse, by absolute values and patterns of normalized capacitance readings. After 24 hours of stabilization, we could obtain stable normalized capacitance values from confluent monolayer of cerebral endothelial cells. In contrast, cerebral endothelial cells which were prepared to be sub-confluent demonstrated further increase in normalized capacitance values to reach the plateau. This characteristic is a distinct one from conventional TEER system, which only can measure the resistance of endothelial cell monolayer in confluent culture condition. Another weakness of TEER is that it strongly depends on the position of the probing electrodes. Furthermore, inherent inhomogeneity of the electrical field across the cell layer typically leads to a systemic overestimation of electrical resistance in TEER system.

A notable result was that capacitance values measured at the low frequency, 100 Hz, were related with paracellular permeability. In contrast, readings at the higher frequency above 75 kHz demonstrated no definite change according to VEGF treatment or the status of confluence. This phenomenon might be due to general properties of dielectric responses of cells and tissues to various ranges of frequency of AC electrical field^10^,^18^. At the low frequency less than few kHz, the electrical current mainly passes through extracellular space; whereas, at the higher frequency between tens of kHz and tens of MHz, it penetrates cell membrane and passes through intracellular space^10^,^18^. Accordingly, capacitance changes at low frequency are known to be related with electrical properties around cellular membrane such as cellular membrane potential and displacement of ions surrounding membranes^10^,^11^,^19^. In lines with these results, our data demonstrated that confluent culture of cerebral endothelial cells were associated with electrical responses of monolayer to low frequency of AC fields, showing potential to be a barometer of the formation and integrity of monolayer.

Another remarkable feature was that we could observe cells directly and optical images could be obtained between measurements. In the research on paracellular permeability, it is advisable to monitor cell morphology during confluent culture for stable maintenance of monolayer characteristics. With additional equipments, live cell imaging can also be a possible option. After successful recording of capacitance values, researchers can perform immunocytochemistry or other molecular works using cells with the references of capacitance measurements.

In summary, this is the first report using dielectric spectroscopy techniques for measuring electrical properties of endothelial cell monolayer to estimate paracellular permeability. Direct measurement of capacitance values at the low frequency enables differentiation of electrical responses of endothelial cell monolayer to a representative permeability-inducing factor, VEGF. In particular, this system effectively captures the status of endothelial cell monolayer, confluent or sparse, which can be utilized as a barometer for stable formation of barrier-like properties. Scalability to live cell imaging, immunocytochemistry, and other assays using cell lysates is also a definite advantage of our capacitance sensor array. We suggest that this system can be a novel *in vitro* method for estimating paracellular permeability of cerebral endothelial cells from capacitance measurement.

## Methods

### Fabrication of capacitance sensors

We fabricated a capacitance sensor array of 16 sensors with interdigitated electrodes on a glass substrate (Fig. 1). By photolithography and lift-off techniques, 100 nm-thickness Au electrodes were patterned with a gap size of 30 μm. A 50 nm-thickness SiO_2_ layer was deposited on the top of Au electrodes to minimize the influence of the cells on the capacitance readings. Then, acrylic wells from Lab-Tek chamber slide (Lot no. 10118584) were attached to the array with a curing agent for the cell culture. All experiments were performed after sterilization of capacitance sensors in an autoclave.

### Cells

bEnd.3 cells (ATCC) were maintained with Dulbecco’s Modified Eagle’s Medium (DMEM; Gibco) supplemented with 10% fetal bovine serum (FBS; Gibco) and 1% Penicillin-Streptomycin (Gibco). ARPE-19 cells (ATCC) were maintained in DMEM/F12 media containing 10% FBS (Gibco) and 1% Penicillin-Streptomycin (Gibco). HRMECs (ACBRI) were maintained in Medium 199 (M199) supplemented with 20% FBS (Gibco), 3 ng/mL basic fibroblast growth factor (Millipore), and 5 IU/mL heparin (Sigma). All cells were incubated in the atmosphere of 95% air and 5% CO_2_ at 37 °C.

### Western blot analysis

Equal amount of proteins extracted from bEnd.3 cells treated of VEGF-165 (20 ng/mL; Cell Signaling) with or without bevacizumab (0.3 mg/mL; Genentech) was separated by SDS-PAGE and transferred to a nitrocellulose membrane. Then, the membranes were incubated with primary antibodies. Primary antibodies were used as follows: zona occludens-1 (ZO-1; Invitrogen) and anti-β-actin (Sigma-Aldrich). Then, the membranes were incubated with species-specific and peroxidase-conjugated secondary antibodies (Thermo) for 1 hour. The visualization of bands was performed with ECL solution (Daeillab) and ImageQuant LAS4000 system (GE).

### Measurement of capacitance

bEnd.3 cells (1 × 10^5^ cells per well for confluent culture; 5 × 10^4^ cells per well for sparse culture) were plated onto each well and incubated for 24 hours in the incubator in the atmosphere of 95% air and 5% CO_2_ at 37 °C. Then, the media were changed with DMEM containing VEGF-165 (20 ng/mL) with or without bevacizumab (0.3 mg/mL). HRMECs (6 × 10^4^ cells per well for confluent culture; 3 × 10^4^ cells per well for sparse culture) were plated onto gelatin (Sigma)-coated well and incubated for 24 hours in the incubator. Then, the media were changed with M199 with 2% FBS containing VEGF-165 (20 ng/mL) with or without bevacizumab (0.3 mg/mL). ARPE-19 cells (7 × 10^4^ cells per well for confluent culture; 3 × 10^4^ cells per well for sparse culture) were plated onto each well and incubated for 24 hours in the incubator. Then, the media were changed with serum free DMEM/F12 containing TNF-α (10 ng/mL; R&D) with or without anti- TNF-α antibody (2 μg/mL; R&D). The concentrations of reagents were selected after the confirmation of no definite cellular toxicity. Capacitance was measured after initial seeding of endothelial cells in wells using Precision Impedence Analyzer (cat. no. 4294A, Agilent) with an AC voltage of 10 mV at various frequencies from 100 Hz to 100 kHz. Data were collected every 5 minutes from each sensor with a data acquisition/data logger switch unit (cat. no. 34970A, Agilent) connected to the impedence analyzer.

### Immunocytochemistry

After the successful recording of capacitance values, the media were removed from each well. Then, the cells were fixated with 4% paraformaldehyde for 15 minutes at room temperature (RT). Permeabilization of cells was performed with 0.1% Triton X-100 in PBS for 15 minutes at RT. Then, to minimize non-specific binding, 3% bovine serum albumin was applied onto cells for 10 minutes at RT. After incubation of cells with antibodies against ZO-1 (cat. no. 339194, Invitrogen) or claudin-5 (cat. no. 352588, Invitrogen) overnight at 4 °C, nuclear staining was performed with 4′,6-diamidino-2-phenylindole (cat. no. D1306, Invitrogen) for 10 minutes at RT. Cells were observed using the confocal microscope after instillation of mounting solution.

### Statistical Analysis

Differences between control and treatment groups were assessed using the 2-tailed unpaired Student’s T-test. All statistical analyses were performed using GraphPad Prism. P-values which were less than 0.05 were considered to be statistically significant. The mean ± SD was shown in figures.

## Additional Information

**How to cite this article**: Hyun Jo, D. *et al*. Real-time estimation of paracellular permeability of cerebral endothelial cells by capacitance sensor array. *Sci. Rep*. **5**, 11014; doi: 10.1038/srep11014 (2015).

## Supplementary Material

Supplementary Information
