# Supplementary material for: Real-time estimation of paracellular permeability of cerebral endothelial cells by capacitance sensor array
Source: Sci Rep. 2015 Jun 5;5:11014. doi: 10.1038/srep11014 (PMC4457143; doi:10.1038/srep11014)
Supplement: Supplementary Information [file srep11014-s1.pdf]

Supplementary Information

Real-time estimation of paracellular permeability of cerebral endothelial cells by capacitance sensor array

Dong Hyun Jo[1,2,5], Rimi Lee[3,5], Jin Hyoung Kim[1], Hyoung Oh Jun[1], Tae Geol Lee[3,*] & Jeong Hun Kim[1,2,4,*]

Affiliations:

[1]Fight against Angiogenesis-Related Blindness (FARB) Laboratory, Clinical Research Institute, Seoul National University Hospital, Seoul, Republic of Korea

[2]Department of Biomedical Sciences, College of Medicine, Seoul National University, Seoul, Republic of Korea

[3]Center for Nano-Bio Measurement, Korea Research Institute of Standards and Science Daejeon, Republic of Korea

[4]Department of Ophthalmology, College of Medicine, Seoul National University, Seoul, Republic of Korea

[5]These authors contributed equally to this work.

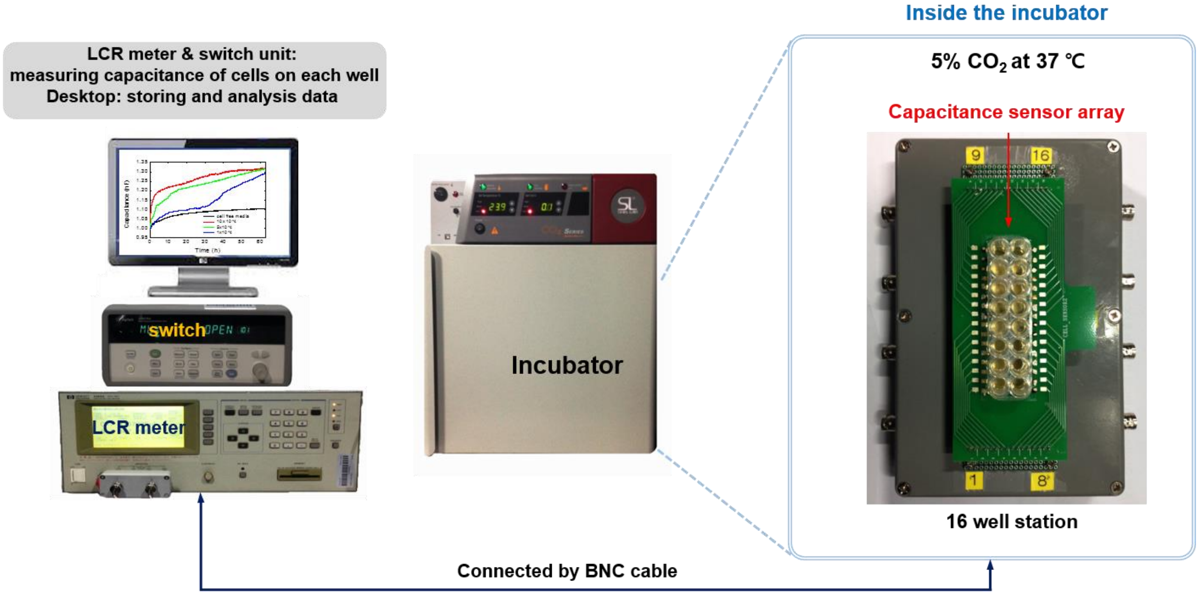

Supplementary Figure 1. A schematic platform of a system of the capacitance sensor array to measure dielectric properties of endothelial cell monolayer. All photographs of each item were taken by one of authors, Rimi Lee by herself.

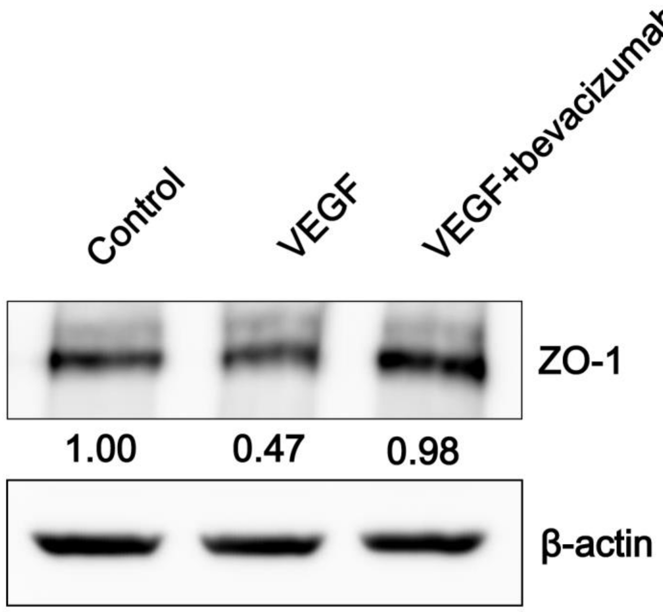

Supplementary Figure 2. Relative expression of ZO-1 in brain microvascular endothelial cells according to the treatment with VEGF and/or bevacizumab. Relative intensity values of bands were provided.

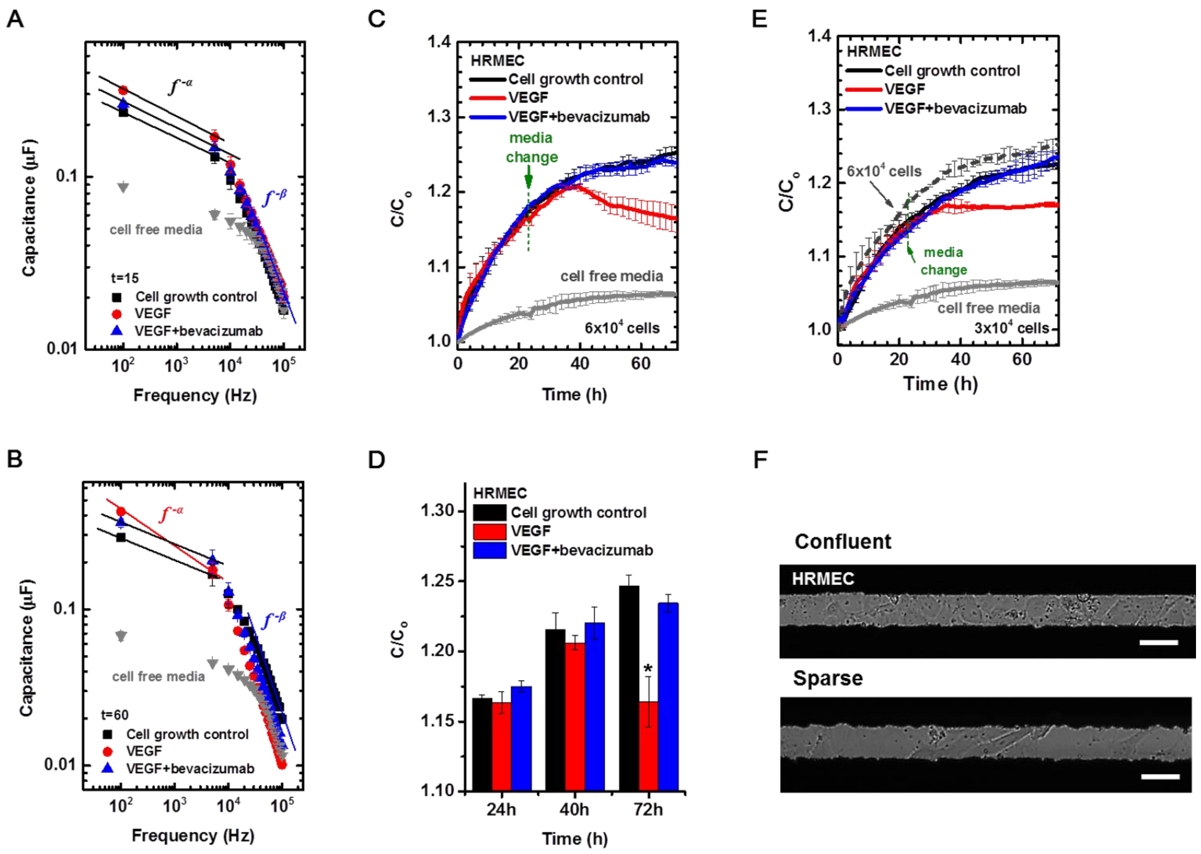

Supplementary Figure 3. Frequency-dependent capacitance measurement in HRMECs. At 15 hours (A) and 60 hours (B) after the initial measurement, capacitance was measured at various frequencies from 100 Hz to 100 kHz (*n* =3). At 15 hours after the initial measurement (before treatment), the capacitance was fitted to the relationship of C ∝ $f^{-\alpha}$ with α ≈ 0.14 for all three groups. In contrast, at 60 hours after the initial measurement (after the treatment with VEGF and/or anti-VEGF antibody), the capacitance followed the relationship ∝ $f^{-\alpha}$ with α ≈ 0.11 for control group and the co-treatment group with VEGF and anti-VEGF antibody, but at α ≈ 0.16 for the group with VEGF treatment at low frequencies. At high frequencies, the capacitance was fitted to C ∝ $f^{-\beta}$ with β ≈ 0.59 at 15 hours and β ≈ 0.61 at 60 hours for all three groups. (C) Time-dependent normalized capacitance values in confluent culture condition. Capacitance values were normalized using C_0, the capacitance measured from cell-free media at Day -1. (D) Bar graph of normalized capacitance values at 20 hours, 40 hours,

and 72 hours after the initial measurement. *, $P < 0.01$ (two-tailed). (E) Time-dependent normalized capacitance values in sparse culture condition. Capacitance values were normalized using C_0, the capacitance measured from cell-free media at Day -1 (F) Optical images of HRMECs in confluent or sparse culture conditions. Scale bars, 30 μm.

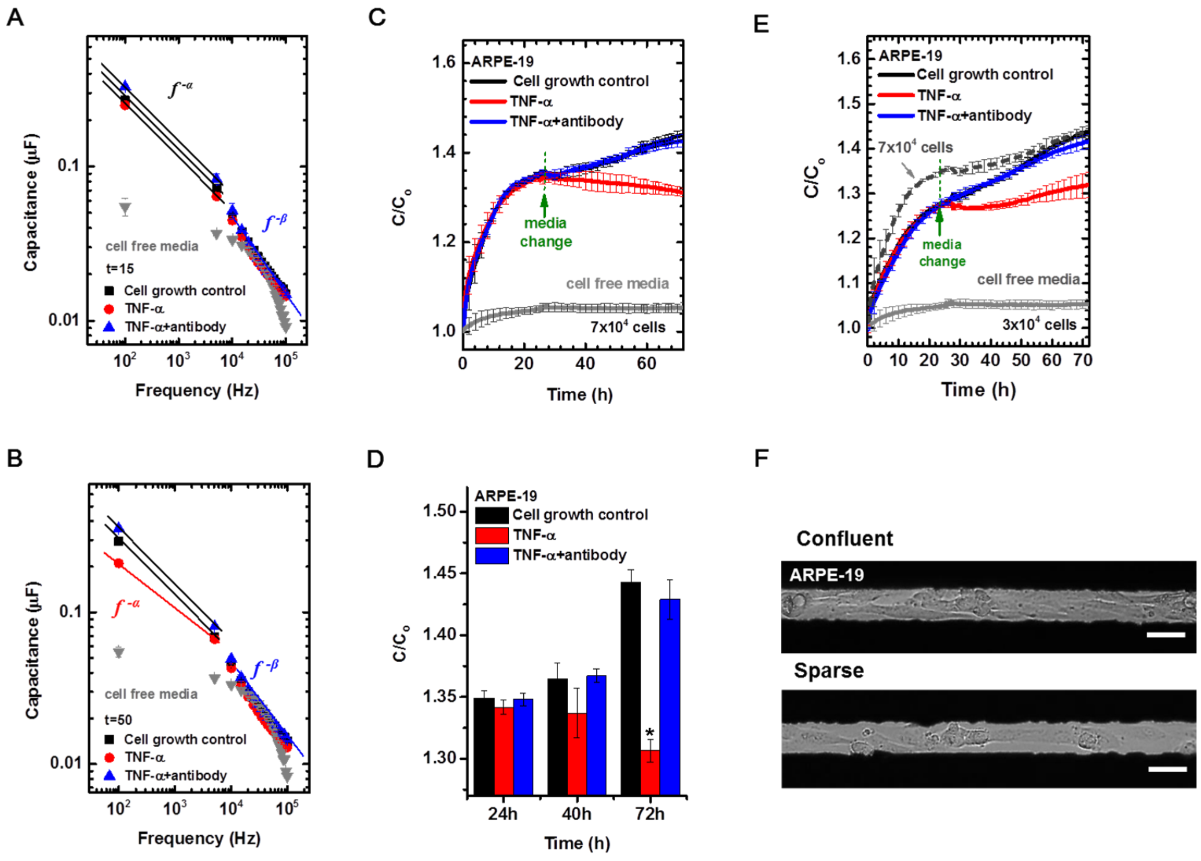

Supplementary Figure 4. Frequency-dependent capacitance measurement in ARPE-19 cells. At 15 hours (**A**) and 50 hours (**B**) after the initial measurement, capacitance was measured from 100 Hz to 100 kHz frequency (*n* =3). At 15 hours after the initial measurement (before treatment), the capacitance was fitted to the relationship of C $\propto f^{-\alpha}$ with $\alpha \approx 0.34$ for all three groups. In contrast, at 50 hours after the initial measurement (after treatment with TNF-α and/or anti-TNF-α antibody), the capacitance followed the relationship $\propto f^{-\alpha}$ with $\alpha \approx 0.37$ for control group and the co-treatment group with TNF-α and anti-TNF-α antibody, but at $\alpha \approx 0.29$ for the group with TNF-α treatment at low frequencies. At high frequencies, the capacitance was fitted to C $\propto f^{-\beta}$ with $\beta \approx 0.41$ at 15 hours and $\beta \approx 0.43$ at 50 hours for all three groups. (C) Time-dependent normalized capacitance values in confluent culture condition. Capacitance values were normalized using C_0, the capacitance measured from cell-free media at Day -1. (D) Bar graph of normalized capacitance values at 20 hours,

40 hours, and 72 hours after the initial measurement. *, $P < 0.01$ (two-tailed). (E) Time-dependent normalized capacitance values in sparse culture condition. Capacitance values were normalized using C_0, the capacitance measured from cell-free media at Day -1 (F) Optical images of ARPE-19 cells in confluent or sparse culture conditions. Scale bars, 30 μm.

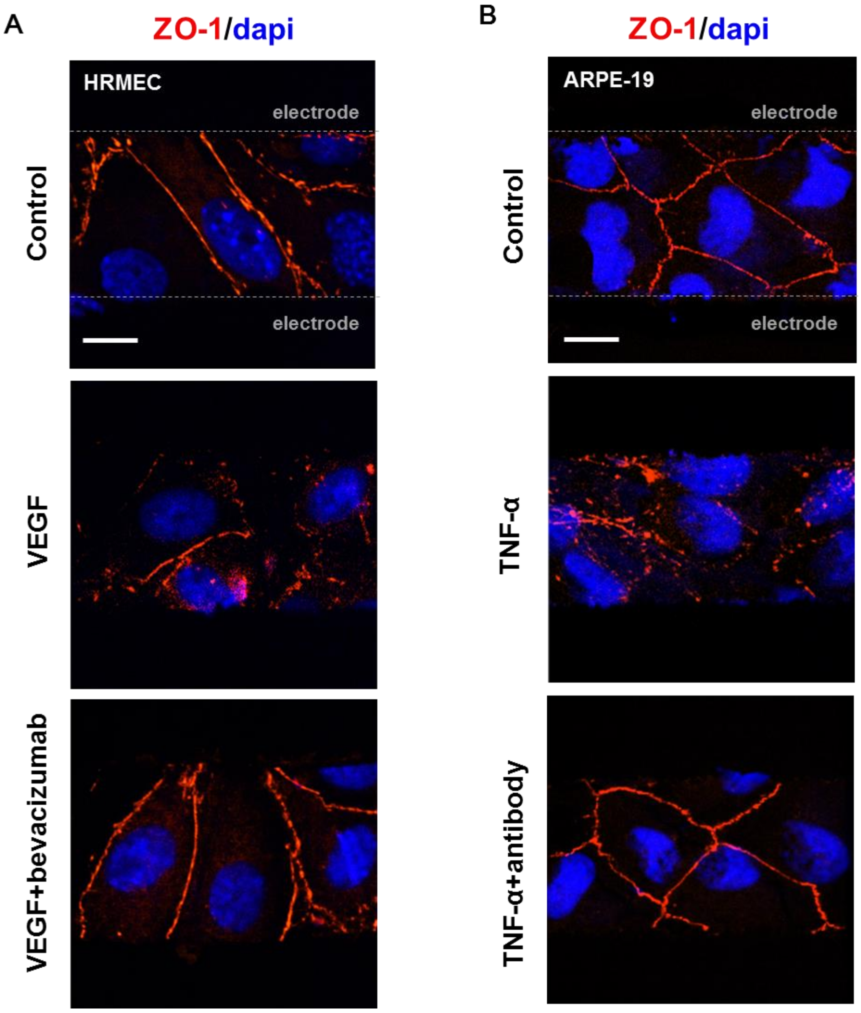

Supplementary Figure 5. Immunocytochemical staining of tight junction proteins in HRMECs and ARPE-19 cells with different paracellular permeability. (A) HRMECs (B) ARPE-19 cells. White arrows indicate disrupted alignment of tight junction proteins. Scale bars, 10 μm.
